# TreeGenes: A Forest Tree Genome Database

**DOI:** 10.1155/2008/412875

**Published:** 2008-08-18

**Authors:** Jill L. Wegrzyn, Jennifer M. Lee, Brandon R. Tearse, David B. Neale

**Affiliations:** ^1^Department of Plant Sciences, University of California, Davis, CA 95616, USA; ^2^Department of Evolution and Ecology, University of California, Davis, CA 95616, USA

## Abstract

The Dendrome Project and associated TreeGenes database serve the forest genetics research community through a curated and integrated web-based relational database. The research community is composed of approximately 2 000 members representing over 730 organizations worldwide. The database itself is composed of a wide range of genetic data from many forest trees with focused efforts on commercially important members of the Pinaceae family. The primary data types curated include species, publications, tree and DNA extraction information, genetic maps, molecular markers, ESTs, genotypic, and phenotypic data. There are currently ten main search modules or user access points within this PostgreSQL database. These access points allow users to navigate logically through the related data types. The goals of the Dendrome Project are to (1) provide a comprehensive resource for forest tree genomics data to facilitate gene discovery in related species, (2) develop interfaces that encourage the submission and integration of all genomic data, and to (3) centralize and distribute existing and novel online tools for the research community that both support and ease analysis. Recent developments have focused on increasing data content, functional annotations, data retrieval, and visualization tools. TreeGenes was developed to provide a centralized web resource with analysis and visualization tools to support data storage and exchange.

## 1. INTRODUCTION

The TreeGenes database is a resource for all forest trees, however work to date has focused on specific members of the Pinaceae family. Pinaceae is one of eight families of the order Coniferales (conifer) and includes 10 genera and approximately 220 species. Species of Pinaceae are commercially important to the forest industry and are used for building, packaging, and paper products worldwide. Because of the very large size and complexity of the conifer genomes (10–40 Gb) [1], greater emphasis has been placed on the expressed portion of the genome. This has been achieved through EST and large-scale resequencing projects. The principal species represented include six members of the genus *Pinus* (*taeda*, *elliottii, radiata, pinaster, sylvestris* and *lambertiana*), two species from other genera of the Pinaceae (*Picea abies* and *Pseudotsuga menzeisii*), and one member of another family of conifer (*Cryptomeria japonica* Cupressaceae). Recent work has also focused on integrating resources for members of Populus and Eucalyptus. Developing genetic resources for these organisms improve the genetic understanding individual member species within the same families or genera. The rapid rate in which genetic data is being generated from large-scale EST and resequencing projects has required corresponding growth in relational databases and associated computational tools. The ability to combine data from different sources facilitates interpretation and potentially allows stronger inferences to be made. When information from several different databases is required, the assembly of data into a format suitable for querying is a challenge. The development of systems for the integration and combined analysis of diverse data remains a priority in bioinformatics. The TreeGenes database helps researchers efficiently analyze, access, integrate, and apply the data. This paper will navigate through the Dendrome (http://dendrome.ucdavis.edu/) and TreeGenes (http://dendrome.ucdavis.edu/treegenes/) interfaces as well as the types of data that can be accessed through these resources.

## 2. CONTENT AND ORGANIZATION

### 2.1. Dendrome project resources

**Figure 1:**
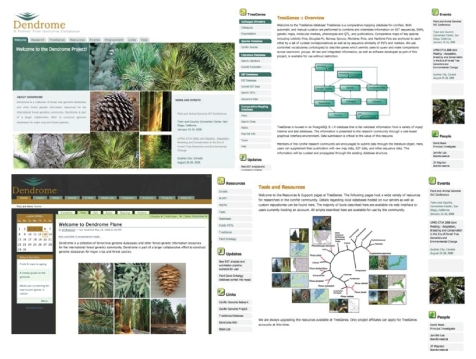
*Diversity of web-based resources available through the Dendrome Project and TreeGenes database*. The Dendrome project (http://dendrome.ucdavis.edu/) serves as a community resource and portal to a variety of resources. These include the TreeGenes database followed by the supporting large-scale project pages, and the Dendrome Plone which is a controlled access forum.

The Dendrome pages are entry points to valuable links, community projects, information forums, custom tools, and the TreeGenes database (http://dendrome.ucdavis.edu/resources/) (Figure 1). The resources available include custom BLAST and FASTA services for sequence similarity searches. These services directly access all publicly available sequence data as well as custom EST or related databases. Users can submit requests for the generation and submission of a searchable database to this repository. An area for the retrieval and submission of custom scripts developed in the community of general use for the manipulation of genetic data exists. This interface allows authors to post and make available scripts and more advanced programs that they have designed including relevant documentation. The *tools* pages are internally curated and provide information on open source and freeware software packages that are relevant to the processing, availability, and analysis of data presented in the TreeGenes database. Links’ pages allow users to submit to and describe online resources and projects relevant to the research community. The links are submitted into one or more relevant genomic categories and are available immediately in the repository.Forms are present through the Dendrome site in order to encourage users to modify, add, and correct a variety of information. The Dendrome pages are the primary source of useful information for the research community such as upcoming events, research opportunities, and the community-curated repository of links. In addition, a discussion forum has recently been implemented to encourage users to submit questions and comments on the database as well as participate in general discussions of conifer genomics. The related Dendrome Plone (http://dendrome.ucdavis.edu/TGPlone), based on the Plone 3.0 content management platform (http://plone.org/), provides a user friendly environment for investigators to share and obtain information relating to a variety of projects.

### 2.2. Treegenes database overview

**Figure 2:**
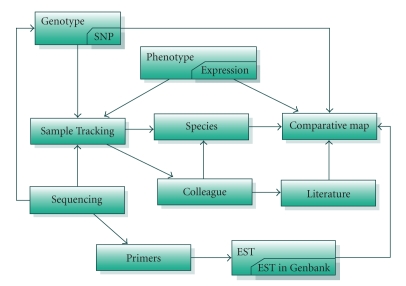
*Modular view of the TreeGenes database schema*. The TreeGenes database (http://dendrome.ucdavis.edu/treegenes/) is a fully relational PostgreSQL database with a total of ten modules. These modules have connections supported by interfaces that allow queries across these data types. Current development is focused on fully incorporating the genotype and phenotype modules.

TreeGenes (http://dendrome.ucdavis.edu/treegenes/) functions through a semi-automated PostgreSQL version 8.1.4-based database that consists of modules to hold a broad range of data and information for trees. This system has a front end consisting of Perl 5.8.5 scripts running in a Linux/Apache/PHP environment. The database is organized into ten different modules that are highly connected in order to ease access and analysis of the data (Figure 2). These modules include sample tracking that holds tree source and DNA extraction information. The sequencing and primers module contains sequences from the resequencing efforts as well as data describing how the sequences were generated and parameters used in their alignment and analysis. The Species module holds the taxonomy information and the Colleague Module contains information on laboratories and individuals. There is a comparative map module that uses Cmap [2] to hold and view genetic maps, map relations, and molecular markers. The Literature module stores publications. The EST module stores sequence and annotation information and ties into an automated pipeline that allows users to submit sequences for processing, analysis, and Genbank submission. TreeGenes is being expanded to include more extensive genotype and phenotype data utilizing data models from the Germinate database [3]. This organization allows for a combination of internal curation as well as third-party submissions to validate and maintain current content.

#### 2.2.1. Species module

Individual species and colleague databases exist in the TreeGenes schema and are well integrated with other database modules. Species is a manually curated database of 222 members of the Pinaceae family. It is currently being expanded to encompass more forest trees. The Species module contains detailed information for each entry including range maps and multiple images for each tree. Internal connections to the researchers who study each species, moreover, relevant publications are available in the detail view. External links to NCBI’s extended taxonomy [4] provide direct access to publicly available sequence sets.

#### 2.2.2. Colleague module

The colleague database is a semi-automated directory of nearly 2 000 researchers and 730 organizations. The colleague interfaces allow users to add, modify, and remove the contact information of both the users themselves and their respective organizations. The interface offers the user the ability to query researchers who focus on particular species in addition to their specific research interests.

#### 2.2.3. Comparative maps module

**Figure 3:**
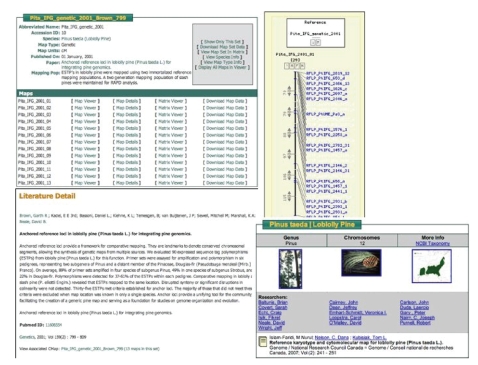
*Comparative mapping (CMAP) interface applied to Pinaceae*. The modified version of Cmap utilized to represent the genetic map unit in TreeGenes is highly interactive. This interface allows for the visualization of comparative map builds including matrix views that display the correspondences between the map sets. Cmap’s internal links also provide detailed supporting information on molecular markers, primer sequences, species, and publications.

The comparative map module is displayed through a modified version of Cmap [2]. Cmap is one component of the larger Generic Model Organism Database (GMOD) toolkit [5]. This package has the ability to compare across genetic, sequence, and physical maps. At this time, TreeGenes only contains genetic maps. The interactive display allows individuals to select single maps and continue to add additional linkage groups from any species to build a comparative view. Maps can be selected by specific linkage groups or they can be queried by their features. TreeGenes currently houses features (marker types) for over 50 genetic maps including AFLP, ESTP, Isozyme, SSR, RAPD, and RFLP. The final map display is highly interactive. Selecting specific features allows for the display of marker information and sequence information. Detail views also include links to internal databases within TreeGenes including species, literature, colleague, EST, QTLs, and PCR primer information (Figure 3). A standardized nomenclature has been developed to describe each potential map feature. Custom scripts exist to generate and enforce this documented nomenclature during the import process. This internal validation allows for name-based correspondences (markers) to be annotated. These correspondences can be easily viewed during comparisons of the maps themselves as well as Cmap’s matrix view.

#### 2.2.4. Literature module

The Literature module is responsible for performing regular and automated searches of major relevant publication repositories including Pubmed [6] and Biosis according to a compiled list of keywords. TreeGenes organizes these publications, creates a list of searchable keywords, and builds internal relations to provide a centralized forest genetics publication repository. This resource can be queried on many levels and allows for a customized subscription to preview recently added publications. In addition to querying public repositories, TreeGenes has a unique feature that allows for the submission of manuscripts by the author. These submitted publications undergo the same process of keyword generation. In addition to the submission of new publications, authors are encouraged to submit supplemental information. Each detail view provides automated external linking in addition to the opportunity to provide sequence data, raw mapping files, or accession numbers from public database submissions. This is the primary mechanism by which the curators can organize the information and further populate the related modules. All standardized nomenclatures are enforced during submission including accessions IDs and TreeGenes comparative mapping nomenclature.

#### 2.2.5. Expressed sequence tags module

**Figure 4:**
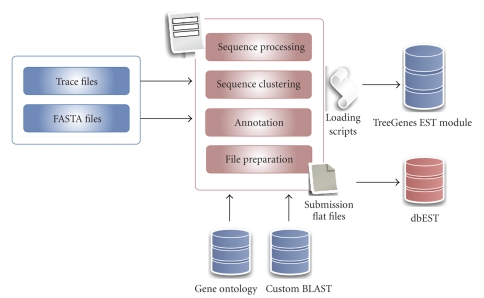
*EST analysis and submission process*. The EST processing and submission pipeline can support multiple projects and species (http://dendrome.ucdavis.edu/treegenes/est/). Tracefiles are renamed, assembled, clustered, annotated, and submitted to Genbank upon the submitter’s approval. The web interface allows users to track the progress, view EST sequences, and provide basic annotation-based searches once the data has been loaded into the TreeGenes database.

The TreeGenes EST pipeline and database apply a combination of custom and open-source tools integrated into a fully automated processing pipeline (Figure 4). The processing occurs at five unique stages and allows users to either original tracefiles or FASTA files. In the latter case, the quality scores are unable to be considered in the processing. (1) Specific EST nomenclature is enforced during submission of the initial trace files or FASTA text files. (2) Tracefiles are processed to identify a filtered, high-quality clone library as determined by Ewing et al. [7]. (3) Sequence clustering consists of assembling high-quality sequences to produce longer transcripts and reduce overall redundancy. This occurs via two rounds of Cap3 [8] processing. (4) Annotation involves pairwise comparisons of the EST clone library and the EST contig consensus sequences. Sequence identification and annotation is provided by a series of BLAST homology searches (Parallel and Priority BLAST) against user-defined and publicly available sequence databases implemented with NCBI’s BLAST [9]. In general, searches are performed against the Genbank nr protein database [4], however users may select custom datasets. The UniGene dataset is derived by selecting the clone that best represents each contig and the singletons that have unique or no matches are further annotated. This level of annotation consists of Gene Ontology (GO) [10] (with preference given to Plant GO [11] hits when available), KEGG for metabolic pathways [12], Enzyme Commission (EC) [13], and InterPro [14] for conserved protein domains (which includes CDD [15], SMART [16], and Pfam [17]). The final stage and optional stage of the processing pipeline involves submission to Genbank [4] following the approval of the owner. Users can login in and view the original EST data, the cleansed data and the analysis results. Tables, formatted text, and links are provided for viewing summary and detailed information. Submission includes the generation, formatting, and actual submission of the required flat files. Since the TreeGenes database is home to many species, the database is organized to maintain independent species and project sets while search interfaces have been developed to support comparative queries.

#### 2.2.6. Resequencing interface

**Figure 5:**
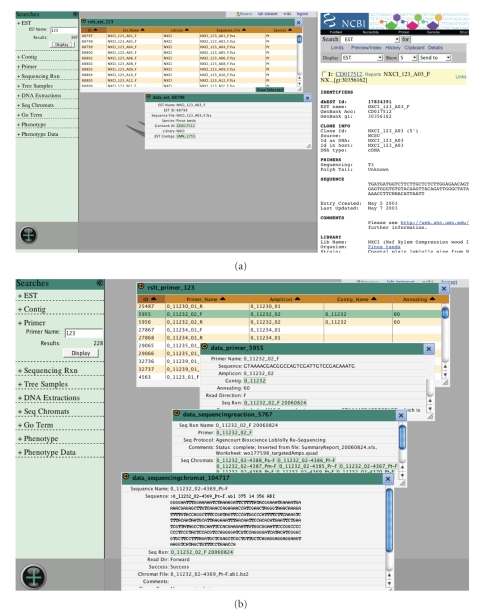
*Resequencing interface*. The workspace interface environment allows for more complex, batch searches of EST, annotation, tracefile, PCR primer, amplicon sequence, SNP, DNA extraction, and tree sample data types (http://dendrome.ucdavis.edu/interface/). Users can initiate searches based on any of these data types. The data retrieved can be compiled into customized lists, saved for future searches, and downloaded to a local machine for further analysis.

The Sample Tracking, Primers, Sequencing, and Genotype modules are currently accessible through an interactive interface that includes a growing library of predefined queries (Figure 5). Each template provides a simplified view of an underlying query by means of a text description and one or more searchable fields. The recent increase in high-throughput sequencing projects encouraged the development of interfaces capable of dealing with the analysis of large amounts of data. In short, researchers can query thousands of sequences in a single operation. The desktop style interface assists with these searches and allows users to customize their results and organize data views. Users can perform multiple searches at one time and combine results. Data types available here currently include ESTs, EST annotation, tracefiles, SNPs, primer sequences, and resequenced amplicons (including DNA extraction and tree sample information) (http://dendrome.ucdavis.edu/interface/). This interface currently accesses information for over 40 000 ESTs and nearly 8 000 resequenced amplicons.

## 3. AVAILABILITY, DATA SUBMISSION, FEEDBACK, AND SUPPORT

The Dendrome Project and the TreeGenes database are publicly available and can be accessed at http://dendrome.ucdavis.edu/. From the website, there is access to help in the form of tutorials and a user manual. We encourage researchers to actively participate in making Dendrome and TreeGenes more accessible by submitting data and providing feedback on general usability. We are interested in being able to provide a unique resource to the community, which is fully dependent on individual submissions. TreeGenes has a robust interface to submit a variety of information including sequence data and comparative mapping files through the literature database. More information on this resource can be found in http://dendrome.ucdavis.edu/TreeGenes/literature/. The discussion forum available through the Dendrome Project website provides an opportunity for users to submit suggestions on improvements or additions to the community resources. Input relating to new functionality and additional data sources are welcome here. A help form is available for user-specific queries. This feedback form will automatically send us the inquiry, making it easier to give an accurate response. Further information is available by joining one of the TreeGenes electronic mailing lists (details on the website) or by email to info@treegenes.ucdavis.edu.

## 4. FUTURE DEVELOPMENT

The structure of TreeGenes permits researchers to rapidly accumulate a wealth of information about a particular object or set of objects. This flexible design facilitates the formulation of new hypotheses for refining subsequent investigations. In addition to refinement and extension of smaller scale investigations, TreeGenes can also facilitate more comprehensive approaches by allowing the investigation of interactions among datasets. TreeGenes is still in a phase of rapid development. Future plans include incorporating standardized gene ontology to describe phenotypic traits unique to forest trees. In addition, robust databases will be available for the submission and visualization of SNP and expression data. The advanced workspace interface will be expanded to accommodate both of these data types as well as tools to ease the analysis and comparison of this information. With an emphasis on meaningful data acquisition and interface design, TreeGenes continues to serve a critical role in the efficient storage and analysis of data by the forest genetics community.

## LIST OF ABBREVIATIONS

EST: Expressed sequence tag
GUI: Graphical user interface
GO: Gene ontology
PHP: Hypertext preprocessor
PO: Plant ontology
SNP: Single nucleotide polymorphism
